# Effects of Maternal Factors on Birth Weight in Japan

**DOI:** 10.1155/2013/172395

**Published:** 2013-11-21

**Authors:** Misato Terada, Yoshio Matsuda, Masaki Ogawa, Hideo Matsui, Shoji Satoh

**Affiliations:** ^1^Department of Obstetrics and Gynecology, Tokyo Women's Medical University, Kawada-cho 8-1, Shinjuku-ku, Tokyo 162-8666, Japan; ^2^Department of Obstetrics and Gynecology, International University of Health and Welfare Hospital, 537-3 Iguchi Nasushiobara, Tochigi 329-2763, Japan; ^3^Maternal and Perinatal Care Center, Oita Prefectural Hospital, Bunyo 476, Oita 870-8511, Japan

## Abstract

*Objective*. We investigated the possible factors related to the birth weight (BW) using the Japanese perinatal database. *Methods*. The live infants born at 37 to 41 weeks of gestation were enrolled in this study. Cases with diabetic pregnancy, preeclampsia, an anomalous fetus, and a fetus with chromosomal abnormalities were excluded. A multiple regression analysis for confounding factors and an analysis of covariance (ANCOVA) for comparing the BW in 2006 and 2010 were used for the statistical analysis. *Results*. The BW significantly decreased from 2950.8 g in 2006 (*n* = 27,723) to 2937.5 g in 2010 (*n* = 38,008) in the overall population, and this decrease was similar for male and female neonates. All confounding factors, except for the mode of delivery, affected the BW. Primiparity, smoking, and a female gender were related to the decrease in BW, whereas maternal age, maternal height, weight gain during pregnancy, BMI, the use of *in vitro* fertilization, induction of labor, and gestational duration were related to an increased BW. The ANCOVA showed that no significant change of the BW was seen between 2006 and 2010 (the difference was 2.164 g, *P* = 0.414). *Conclusion*. The gestational duration is the most important factor affecting the BW in singleton term infants.

## 1. Introduction

There is a widespread belief that there has been an increase in the babies' birth weight (BW) [1, 2] whereas there has been a decrease in the BW in Japan [3, 4]. The average BW has fallen by 125 g in the past 25 years [5]. Low birth weight (LBW) in infants has been linked to not only an increased risk of neonatal mortality and morbidity, physical and psychomotor developmental delays, and an increased rate of significant disabilities, but also to long-term problems, such as developmental origin health and disease (DOHaD) [6, 7]. Even in adult life, LBW predisposes subjects to chronic diseases, such as ischemic heart disease and diabetes.

However, the surveillance data reported above included preterm births and multiple gestations and did not account for BW in maternal or neonatal characteristics or obstetric practices in detail [8]. It has also been unclear what factors have contributed to the neonatal BW.

Therefore, we performed this study to investigate the possible factors related to the BW in Japan, especially among singleton live infants born at term, using the Japanese perinatal database (JPDB). 

## 2. Materials and Methods

The study protocol was reviewed and approved by the Ethics Committee of Oita Prefecture Hospital. Detailed descriptions of the database have been published previously [9, 10]. In brief, a self-administered questionnaire, interview, and medical records were used to collect information on the parity, maternal age at delivery, maternal height, body mass index (BMI) before pregnancy, smoking habit, alcohol intake during pregnancy, medical history, history of treatment for infertility, major obstetric complications during pregnancy, weight gain during pregnancy, mode of delivery, infant sex, gestational length (weeks), induction of labor, and mode of delivery. Data entry was routinely performed by attendants at the time of delivery. The data conform to uniform coding specifications and diagnostic criteria for complications and were subject to rigorous quality checking. The dataset for the study was provided by the Japan Society of Obstetrics and Gynecology, where the quality control for the database was assessed. Thereafter, the data were edited and reviewed. 

We restricted our analysis to patients who delivered a single live infant between 37 and 41 weeks of gestation and excluded those for whom data were unavailable. Only term deliveries were included to avoid any effect of the increasing number of preterm births on the mean BW.

The gestational age was determined based on the menstrual history, the prenatal examination, and ultrasound findings during early pregnancy (gestational sac diameter, crown rump length, and biparietal diameter). 

As confounding factors, the maternal age at delivery, maternal height, body mass index (BMI) before pregnancy, smoking habit, use of *in vitro* fertilization, weight gain during pregnancy, infant sex, the parity, gestational length (weeks), induction of labor, and mode of delivery were included. 

In order to avoid perinatal/obstetrical factors affecting fetal growth, patients with pregestational/gestational diabetes, who had anemia, preeclampsia, an anomalous fetus, or a fetus with chromosomal abnormalities, were excluded [8, 11, 12].

The statistical analyses were performed using the SAS 9.1 software program (SAS Institute, Cary, NC, USA). The results were expressed as the means ± SD. Student's *t*-test for continuous variables and the chi-square test for categorical variables were used. To investigate the association between the BW and the proposed explanatory variables, we used multiple regression analysis on the 2006 and 2010 data, because all variables listed above were included in these data, and also performed an analysis of covariance (ANCOVA) for the change, with the significance level set at <0.05.

## 3. Results

The general characteristics of the survey participants according to survey year are shown in Table 1. The change in infants was characterized by a significant decrease in the mean BW from 2950.8 g in 2006 (*n* = 27,723) to 2937.5 g in 2010 (*n* = 38,008), and this decrease was similar for male and female infants. 

Comparing the factors between 2006 and 2010, the gestational age at delivery was increased and smoking was more frequent in 2006, whereas the maternal height, pregestational body weight, pregestational BMI, use of IVF-ET, primiparity, induction of labor, and rate of cesarean section delivery were higher in 2010. 

In order to identify factors that could explain the decrease in the birth weight between the two recent survey periods (2006 and 2010), we applied a multiple regression analysis using the datasets from the 2006 and 2010 surveys, as shown in Table 2. All confounding factors except the mode of delivery affected the BW. 

Primiparity (−105 g), smoking (−108 g), and female gender (−107 g) were all related to the decrease in BW, whereas the maternal age (+2.8 g/year), maternal height (+10.3 g/cm), weight gain during pregnancy (+16.3 g/kg), BMI (+22.3 g/index), use of *in vitro* fertilization (+37.4 g), induction of labor (+9.7 g), and gestational duration (+160 g/week) were associated with increases in the BW. 

Using ANCOVA, we calculated the difference of BW in order to clarify the effect of each confounding factor, and it has been shown that no significant change in the BW was seen between 2006 and 2010 after adjusting each factor (the difference was 2.164 g, *P* = 0.414) (Table 3).

## 4. Discussion

The data from the JPDB in 2006 and 2010 were used to identify the underlying factors influencing BW in Japan and to investigate the current risk factors for BW. The present study showed data that were consistent with prior investigations regarding the relationship between the BW and perinatal/obstetrical factors [3, 4]. Among singleton pregnancies, a shorter gestational length (duration), female infant, maternal primiparity, and smoking have previously been demonstrated to be the main factors for BW. Particularly, the gestational duration mostly affected the BW, because this net effect of BW (16.36 g) was the highest in all confounding factors (Table 3). A reduction of 13 g in BW may not matter for an individual infant but represents a substantial change for an overall population. It is unclear why gestational age at birth is reducing in Japan. It may be a result of the increase in planned delivery before the due date with a doctor shortage and the increase in complicated pregnancy.

Although there has been an increase in the infants BW in the past, this upward trend in BW has more recently been reversing [13]. For example, from 1990 to 2005, the BW decreased among term births in the United States, especially after 1999 [8, 14].

The Children and Infant Growth Survey is a national Japanese survey on anthropometric parameters (weight, height, head circumference, and chest circumference) in children between birth and six years old, and it has been carried out every 10 years since 1950. Information on the birth and maternal background has also been recorded in this survey [15]. Using this database consisting of randomly selected population-based surveys, Takimoto et al. reported the prevalence of LBW infants in Japan from 1980 to 2000 [4]. They showed that the proportion of LBW infants was 4.2% in 1980, 6.1% in 1990, and 8.3% in 2000, and that the mean BW were 3,189 g in 1980, 3,123 g in 1990, and 3,033 g in 2000, which were compatible to corresponding vital statistics reports (3,190 g in 1980, 3,080 g in 1990, and 3,030 g in 2000, resp.), and the increase in preterm deliveries and multiple gestations were found to be important factors with regard to the increase in LBW infants in Japan. Ohmi et al. hypothesized that a decrease in BMI in young females could be related to the increase in LBW infants [3]. Indeed, it has been demonstrated that the increase in nutritionally derived underweight females with an insufficient diet while pregnant has led to poor maternal weight gain and to affecting optimal fetal growth [4]. 

Although they did not demonstrate a strong effect of smoking and maternal age on the LBW increase, we have shown a significant correlation between them in the present study. Other important factors influencing pregnancy outcomes, such as the weight gain during pregnancy [16, 17], use of assisted reproductive technologies (ART) [18], pregnancy complications [11], induction of labor [14], and mode of delivery [19, 20], were also assessed in this survey.

As a result, we found the maternal height, pregestational BMI, weight gain during pregnancy, use of ART, and induction of labor to all have a positive effect on the BW. Because information on the medical indications for the induction of labor could not be obtained from the database, we could not explain the potential cause of this increase. 

In brief, we have herein extracted the maternal and neonatal characteristics associated with fetal growth and gestational age as possible factors related to the infant BW. This is a major strength of this study. For example, Kramer et al. reported temporal trends in fetal growth. Although their report was a hospital-based study in Canada, various pieces of information, such as the parity or use of ART, were lacking [21]. In Lehmann's report from Australia, the maternal height, weight gain during pregnancy, smoking, use of ART, and mode of delivery were all unknown [22]. Second, we assessed the gestational duration as gestational days, not in gestational weeks. Although the gestational age at birth is generally reported in completed weeks, it is possible that a decrease of gestational length of a few days within each gestational week might account for the observed decline in fetal growth. 

Despite the fact that this analysis was based on a large number of subcohorts of pregnancies, some limitations of this study merit attention. First, our data was limited to information derived from discharge record abstracts. The second shortcoming of our study is the use of a database, in which coding errors are known to occur. Other factors not recorded in a database that might contribute to the decline in gestational length or fetal growth include trends in maternal physical activity, stress, socioeconomic factors, pollution or toxicant exposures, or unrecorded medical conditions, such as asthma or thyroid disease. More detailed studies of smaller populations would be needed to explore the role of these factors [8]. Third, because we examined an annual change in BW in only two time points, we could not show yearly trends, although there was a reduction in BW by about 15 g. 

In conclusion, we have demonstrated that the difference of BW of babies born at term in Japan was explained by maternal factors and the difference of maternal background. Further studies to ascertain all factors contributing to the decrease in BW over time, including other factors that might contribute to declines in fetal growth, are warranted. In addition, future study is also needed whether the prediction of the annual trends is possible, considering the chronological change in obstetrical background. 

## Figures and Tables

**Table 1:** Comparison of the perinatal/obstetrical variables between 2006 and 2010: results of the univariate analysis (total).

	2006	2010	*P*
Number	27,723	38,008	
Birth weight (g)	2950.84 (510.24)	2937.52 (505.12)	<0.0001
Gestational age at delivery (wks)	39.087 (1.933)	38.985 (1.930)	<0.0001
Maternal height (cm)	158.338 (5.436)	158.471 (5.492)	0.006
Maternal age (yr)	31.119 (5.015)	32.002 (5.209)	0.006
Pregestational BMI	21.057 (3.347)	21.092 (3.44)	0.041
Weight gain during pregnancy (kg)	9.846 (4.454)	9.842 (4.333)	0.744
IVF-ET	637 (2.3%)	1,672 (4.4%)	<0.0001
Primiparity	14,693 (53.0%)	20,676 (54.4%)	<0.0001
Smoking	1,807 (5.3%)	1,672 (4.4%)	<0.0001
Induction of labor	6,542 (23.6%)	10,224 (26.9%)	<0.0001
Cesarean delivery	6,986 (25.2%)	10,832 (28.5%)	<0.0001

() represents the standard deviation or %.

**Table 2:** Factors affecting the birth weight as determined by a multiple regression analysis (total).

	Partial regression coefficient	*P*
Gestational age at delivery (/wks)	160.081 (158.141–161.171)	<0.0001
Primiparity (yes versus no)	−104.796 (−112.232–−101.248)	<0.0001
Cesarean delivery (yes versus no)	−2.046 (−7.938–6.019)	0.787
Weight gain during pregnancy (kg)	16.269 (15.479–16.741)	<0.0001
Female gender	−107.150 (−112.585–−102.231)	<0.0001
Induction of labor (yes versus no)	9.714 (5.483–18.734)	<0.0001
Pregestational BMI (/1)	22.250 (21.264–22.875)	<0.0001
IVF-ET (yes versus no)	37.404 (17.161–46.538)	<0.0001
Smoking habit (yes versus no)	−107.593 (−118.235–−93.763)	<0.0001
Maternal height (/cm)	10.270 (9.908–10.864)	<0.0001
Maternal age (year)	2.752 (2.117–3.188)	<0.0001

**Table 3:** Differences of birth weight between 2006 and 2010 before and after adjusting each confounding factor by ANCOVA.

	Value		Difference			
	2006	2010				
Difference of birth weight before adjustment by ANCOVA						
Birth weight (g)	2950.840	2937.520	13.320	—	—	13.320

Estimation for net effect on birth weight of each factor by ANCOVA						
Partial confounding factors				b*	Net effect (g)^†^	Total effect (g)^‡^

Gestational age at delivery (weeks)	39.087	38.985	0.102	160.081	16.360	11.156
Primiparity	0.530	0.544	−0.014	−104.796	1.496	
Cesarean delivery	0.236	0.285	−0.050	−2.046	0.102	
Weight gain during pregnancy (kg)	9.846	9.842	0.004	16.269	0.064	
Female gender	0.489	0.487	0.002	−107.150	−0.236	
Induction of labor	0.236	0.269	−0.033	9.714	−0.322	
Pregestational BMI	21.057	21.092	−0.035	22.250	−0.774	
IVF-ET	0.023	0.044	−0.021	37.404	−0.785	
Smoking habit	0.053	0.044	0.009	−107.593	−0.954	
Maternal height (cm)	158.338	158.471	−0.133	10.270	−1.366	
Maternal age (year)	31.119	32.002	−0.883	2.752	−2.428	

Difference of birth weight after adjustment by ANCOVA						
Birth weight (g)						2.164

*b meant a partial regression coefficient. ^†^Net effect was calculated by product of difference and partial regression coefficient. ^‡^Total effect was derived from sum of net effect.

ANCOVA: analysis of covariance, BMI: body mass index, and IVF-ET: *in vitro* fertilization and endometrial transfer.
